# Semantic Particularity Measure for Functional Characterization of Gene Sets Using Gene Ontology

**DOI:** 10.1371/journal.pone.0086525

**Published:** 2014-01-28

**Authors:** Charles Bettembourg, Christian Diot, Olivier Dameron

**Affiliations:** 1 Université de Rennes 1, Rennes, France; 2 UMR1348 PEGASE, INRA, Saint-Gilles, France; 3 UMR1348 PEGASE, Agrocampus OUEST, Rennes, France; 4 IRISA, Campus de Beaulieu, Rennes, France; 5 INRIA, Rennes, France; The Centre for Research and Technology, Hellas, Greece

## Abstract

**Background:**

Genetic and genomic data analyses are outputting large sets of genes. Functional comparison of these gene sets is a key part of the analysis, as it identifies their shared functions, and the functions that distinguish each set. The Gene Ontology (GO) initiative provides a unified reference for analyzing the genes molecular functions, biological processes and cellular components. Numerous semantic similarity measures have been developed to systematically quantify the weight of the GO terms shared by two genes. We studied how gene set comparisons can be improved by considering gene set particularity in addition to gene set similarity.

**Results:**

We propose a new approach to compute gene set particularities based on the information conveyed by GO terms. A GO term informativeness can be computed using either its information content based on the term frequency in a corpus, or a function of the term's distance to the root. We defined the semantic particularity of a set of GO terms Sg1 compared to another set of GO terms Sg2. We combined our particularity measure with a similarity measure to compare gene sets. We demonstrated that the combination of semantic similarity and semantic particularity measures was able to identify genes with particular functions from among similar genes. This differentiation was not recognized using only a semantic similarity measure.

**Conclusion:**

Semantic particularity should be used in conjunction with semantic similarity to perform functional analysis of GO-annotated gene sets. The principle is generalizable to other ontologies.

## Introduction

With the continued advance of high-throughput technologies, genetic and genomic data analyses are outputting large sets of genes. The amount of data involved requires automated comparison methods [1]. The characterization of these sets typically consists in a combination of the following three operations [2], [3]: first, synthesize the over- and under-represented functions of these genes [4], [5]; second, identify how these genes interact with each other [6]; third, identify and quantify the common shared features and the differentiating features [7], [8]. A widely used method for genes sets study called “Gene Set Enrichment Analysis” (GSEA) determines which gene features are over-represented in a gene set [9]. Numerous tools have been developed in this purpose: BiNGO [10], GOEAST [11], ClueGO [12], DAVID [13], GeneWeaver [14], GOTM [15]. See Hung et al. recent work for a review [16]. GSEA is useful for clustering a set of genes into subsets sharing over-represented features. Among these features, the biological processes (BP), molecular functions (MF) and cellular components (CC) annotating each gene are represented using the Gene Ontology (GO) [17]. GO is species-independent, and thus supports cross-species comparison [18]. The GO graph itself is also widely used for genes semantic similarity analysis [19].

### Semantic similarity

Within a given gene set, the genes sharing identical or similar GO annotations can be grouped into clusters using two approaches [20]. The GSEA approach computes these clusters considering the GO terms over-representation. The semantic similarity approach takes into account GO properties to cluster genes considering the quantity and the importance of their shared annotations [21]–[24]. Both approaches are not exclusive, as semantic measures can be involved in GSEA in order to improve the analysis [25]. If these terms were independent, the gene set characterization could be performed by a straightforward set-based approach such as the Jaccard index or Dice's coefficient. However, GO terms are hierarchically-linked. Consequently, the characterization needs to take into account the underlying ontological structure of the GO annotations [26].

Semantic similarity measures rely on ontologies to systematically quantify the weight of the shared elements. They exploit the formal representation of the meaning of the terms by considering the relations between the terms (e.g. for inferring new annotations that were implicit as each term inherits all the properties of its ancestors) and by attributing different weights to each term depending on how much information they convey. When working with annotation databases, it should be routine practice to use the ontology hierarchy to infer implicit annotation [26]. Pesquita et al. performed an extensive review of the main semantic similarity measures [27] and identified two main categories, i.e. node-based methods and edge-based methods, as well as a handful of hybrid methods.

Node-based semantic similarity measures rely on how informative the terms are. Typically, they consider that two terms sharing an informative lowest common ancestor are more similar than two terms with a less informative lowest common ancestor. Historically, Information Content (IC) value was used to quantify how informative a term is, with the least frequent terms having the highest IC value. This concept, borrowed from Shannon's Information Theory [28], was used to measure similarities using ontologies [29]–[31] such as WordNet [32]. To compare two terms, these methods rely on their most informative common ancestor (MICA). The IC of this ancestor is the semantic similarity value between the compared terms. These methods developed in linguistics have been applied to GO [33], [34] using the frequency with which a term annotates a gene as a marker of its rarity. Consequently, the IC of a GO term is inversely proportional to the frequency with which it annotates a gene using the Gene Ontology Annotations (GOA) database [35]. GOA specifies also how each annotation has be attributed through Evidence Codes (EC). In their method called “IntelliGO”, Benabderrahmane et al. use a weighting corresponding to each GO term EC in addition to their IC [36]. Retrieving only the most informative common ancestor to compute a semantic similarity ignores the possibility that two GO terms can share several common ancestors. These situations result in a loss of information. A possible solution has been proposed that consists in using the average of the IC values of all disjoint common ancestors (DCA) instead of the maximum IC of this common set [37]. For the node-based methods relying on IC, the terms' frequencies used to compute the IC values depend on the corpus of reference. In the context of genes comparison, IC-based methods have three main limits related to their dependence on a GOA-based corpus. First, it can prove difficult or even impossible to obtain a relevant corpus. GOA provides single and multi-species annotation tables. Although using a species-specific table is well-suited to intra-species comparisons, it becomes problematic for cross-species comparisons. Second, using a multi-species table (like the UniprotKB table) in these cases is biased towards the most extensively annotated species such as human or mice. Third, the well-studied areas of biology have high annotation frequencies and are therefore less informative and see their importance downgraded, whereas the less-studied areas are artificially upgraded [38]–[40].

Edge-based semantic similarity measures use the directed graph topology to compute distances between the terms to compare. Rada distance is based on the shortest path between the two terms [41]. Such distances rely on the average path among multiple paths [27]. Other approaches take into account the length of the path between the root of the ontology and the least common ancestor (LCA) of the terms, with the result that terms with a deep common ancestor are more similar than terms with a common ancestor close to the root [42]–[46]. The edge-based methods using depth as a proxy for precision are not dependent on a particular corpus. This can be a good thing when it is difficult or impossible to determine a representative corpus, or a bad thing when corpus-dependent frequencies are relevant. Moreover, another constraint to consider is that granularity is not uniform in GO, so terms at the same depth can have different precisions [47].

Pesquita et al. also identified “hybrid” methods that combine different aspects of node-based and edge-based methods. In Wang's method [22], each term has a “semantic value” that represents how informative the term is, conforming to the node-based approach. However, the semantic value of a term is obtained by following the path from this term to the root and summing the semantic contributions of all the ancestors of this term. As the semantic value depends on the ontology topology, it also conforms to the edge-based approach.

Pesquita et al. do not single out any particular semantic similarity measure as the best one, as the optimal measure will depend on the data to compare and the level of detail expected in the results. The main advantage of Wang's method compared to purely node-based methods is that the semantic value is not GOA-dependent, unlike information content. It is thus well-suited to cross-species comparisons. As cross-species comparison is one of the key stakes in biology, further development in the domain of semantic comparison should support such comparisons.

### Limitations of semantic similarity

**Figure 1:**
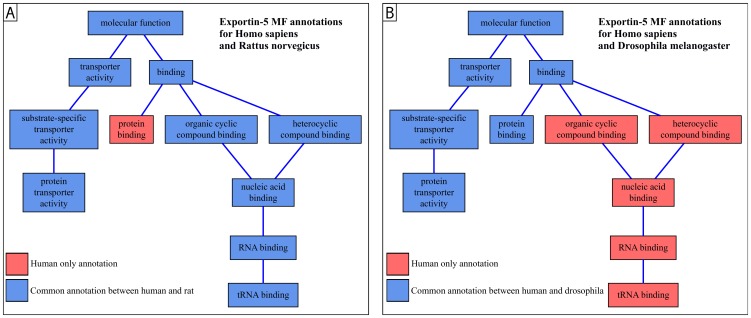
Representation of Exportin-5 orthologs annotations. Common terms between species are displayed in blue. The terms annotating only the human ortholog are displayed in red. Part A of this figure displays the MF annotations of the human and rat orthologs of Exportin-5. Part B displays the MF annotations of the human and drosophila orthologs of Exportin-5. In this example, there is no rat nor drosophila-specific term. The semantic similarity values obtained in these cases do not reflect the difference of human particularity between each part.

All the semantic similarity measures appear appropriate for identifying and quantifying common features. However, as these measures are focusing on common features, they may lead to an incomplete analysis when comparing genes having particular features along side similar ones [48]. For example, parts A and B of Figure 1 respectively present the MF terms annotating the Exportin-5 orthologs of human (hsa) and rat (rno) and the Exportin-5 orthologs of human and drosophila (dme). Wang's method allows to compute cross-species semantic similarity. The results on MF annotations are: Sim(hsa, rno) = 0.797 and Sim(hsa, dme) = 0.726. This is consistent with the fact that globally, the Exportin-5 orthologs share the same functions between hsa, rno and dme. However, there are also five times as many human-specific MF terms compared to drosophila as compared to rats. It has been demonstrated that Exportin-5 orthologs are functionally divergent among species [49]. The tiny difference of semantic similarity (0.071) correctly reflects the fact that the orthologs share the same main function, but is not sufficient to identify that some species also have additional functions.

We assume that considering only similarity measures is not enough to compare sets of annotations. This analysis is valid for any set of annotations that refer to an ontology. We hypothesize that gene set analysis can be improved by considering gene particularities in addition to gene similarities. We propose a general definition and some associated formal properties. We propose also a new approach based on the notion of GO term informativeness to compute gene set particularities.

## Methods

### Definition of semantic particularity

The semantic particularity of a set compared to another is the value that reflects the importance of the features that belong to the first set but not the second. To compare two genes, we rely on the similarity and the respective particularities of their sets of annotations. The particularity of a gene g1 annotated by the set Sg1 compared to a gene g2 annotated by the set Sg2 depends on the annotations of Sg1 that are not related to any annotation of Sg2.

### Formal properties

Like for semantic similarity, we compute a value bounded by 0 (least particular) and 1 (most particular). Four important properties arise from the semantic particularity definition:

- The semantic particularity is non-symmetric:

Par(Sg1, Sg2) = x⇏Par(Sg2, Sg1) = x **(Prop 1)**

- Compared to itself, a set of annotations has no semantic particularity:

Par(Sg1, Sg1) = 0 **(Prop 2)**

If Sg1 = 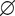, this comparison is meaningless.

- The semantic particularity of a set of annotations Sg1 (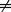 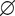) is maximal when it is compared to an empty set of annotations:

Par(Sg1, 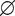) = 1 **(Prop 3.1)**

And conversely:

Par(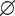, Sg1) = 0 **(Prop 3.2)**

- The particularity of a set Sg1 of annotations compared to a set Sg2 does not depend on the elements of Sg2 that do not belong to Sg1:

Sg3
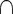
Sg1 = 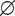 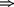
Par(Sg1, Sg2) = Par(Sg1, Sg2
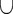
Sg3) **(Prop 4)**

### Measure of semantic particularity

In order to compute the particularity of Sg1 compared to Sg2, we focus on the terms of Sg1 that are not members of Sg2. This requires to address two problems: the terms are not independent, and they do not convey the same amount of information.

Some of the terms of Sg1 that are not members of Sg2 may be linked in the graph. Taking several linked terms into account would result in considering them several times. For example, in Figure 1B, considering both “RNA binding” and “tRNA binding” would result in counting twice the contribution of “RNA binding”. Therefore, we should only focus on the terms of Sg1 that do not have any descendant in Sg1 and that are not members of Sg2. Some of these terms might be ancestors of terms of Sg2 and should be considered as common to Sg1 and Sg2. We call Sg^*^ the union of Sg and the sets of ancestors of each element of Sg. We call MPT(Sg1, Sg2) the set of most particular terms of Sg1 compared to Sg2. MPT(Sg1, Sg2) is the set of terms of Sg1 that do not have any descendant in Sg1 and that are not members of Sg2^*^. In the Figure 1B, MPT(hsa, dme) = [“tRNA binding”].

Using the set theory, we could define Par(Sg1, Sg2) as the proportion of elements of Sg1 that belong to MPT(Sg1, Sg2). When computing card(MPT(Sg1, Sg2)), all the elements have the same weight. However, considering the semantics underlying these elements, some of them may be more informative than others and should ideally be emphasized. Different strategies, similar to those already proposed for the computation of the semantic similarity, can be applied.

We then define PI(Sg1, Sg2), the particular informativeness of a set of GO terms Sg1 compared to another set of GO terms Sg2, as the sum of the differences between the informativeness (I) of each term 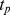 of MPT(Sg1, Sg2) and the informativeness of the most informative common ancestor (MICA) between 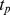 and Sg2. The PI of a set of terms is the information that is not shared with the other set.
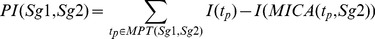
(1)


In the Figure 1B, PI(hsa, dme) = I(tRNA binding)−I(binding). We have no sum in this example since MPT(Sg1, Sg2) only contains one term.

We last normalize PI to compute Par(Sg1, Sg2), the semantic particularity of the set of GO terms Sg1 compared to the set of GO terms Sg2. We define MCT(Sg1, Sg2), the set of the most informative common terms of Sg1 and Sg2, as the set of the terms belonging to the intersection of Sg1^*^ and Sg2^*^ that do not have any descendant either in Sg1^*^ or in Sg2^*^. In the Figure 1B, MCT(hsa, dme) = [“protein transporter activity”, “protein binding”]. Par(Sg1, Sg2) is the ratio of PI(Sg1, Sg2) and the sum of the informativeness of Sg1 most informative terms (i.e. those Sg1-specific and those common with Sg2; the MICA in the PI formula for the Sg1-specific guarantees that the informativeness of common terms is not counted twice).
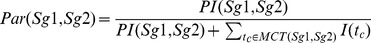
(2)


For the example of the Figure 1B, this formula becomes:
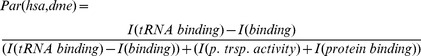
(3)


Several measures of informativeness have been proposed. The widely used Information Content (IC) family depends on an annotation corpus (e.g. GOA). The IC of a term t is its negative log probability 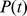.
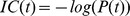



In the context of GO terms comparison, the probability of occurrence of a term 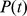 is estimated by its frequency in annotations [27]. It is necessary to take into account Gene Ontology subsumption hierarchy when computing this frequency in order to also consider implicit annotations to the terms descendants [26]. IC is typically used when a representative corpus is available such as human GOA for studying human genes functions.

The alternative approach is corpus-independent. A term informativeness is a function of its distance to the root. It is typically used when a relevant corpus cannot be computed (for comparing elements from several species) or does not exist (for poorly studied species). Wang's Semantic Value (SV) computes this type of informativeness. The relevance of the results obtained by this approach has previously been demonstrated [22], [27]. Wang first computes the semantic contributions of the ancestors of each term to compare to these terms, following:
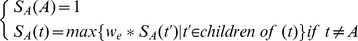
where S*_A_* (t) is the semantic contribution of the term t to the term A and w*_e_* is the semantic contribution factor for edge e linking a term t with its child term t'. According to Wang, we use a semantic contribution factor of 0.8 for the “is a” relations and 0.6 for the “part of” relations, and we added a 0.7 factor for the “[positively] [negatively] regulates” relations. An additional study not presented here showed that the value of the regulation factor had minimal impact (+/−0.01) on the overall value.

Then, for each target term to compare, the semantic value is the sum of the semantic contributions of all its ancestors:
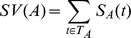



As shown in the equation 3, four terms are involved in the calculation of the MF particularity of the human Exportin-5 ortholog compared to the drosophila Exportin-5 ortholog. This comparison is cross-species, so a semantic value-based informativeness measure is relevant. According to the previous formula, the semantic values of the terms involved in the equation 3 are: SV(tRNA binding) = 4.201, SV(binding) = 1.8, SV(protein transporter activity) = 2.952 and SV(protein binding) = 2.44. Consequently, we can compute: Par(hsa, dme) = 0.308. Likewise, for Figure 1A, Par(hsa, rno) = 0.082.

## Results

To study the benefits of our approach over an analysis based only on similarity, we considered three biological cases. In order to determine if we could extend Wang's initial results, our first use case was *Saccharomyces cerevisiae* tryptophan degradation. As both the ontology and the annotations have evolved since 2007 [39], we computed the updated semantic similarity. Then, we computed the particularity measure in order to evaluate its benefits. In case 2, we computed the similarity and particularity values on a set of 51 gene products belonging to a same human metabolic pathway. The motivation is to study whether the results of the case 1 can be generalized to a larger set of genes. We also studied how using IC-based or semantic value-based similarity and particularity measures affects the conclusions. In case 3, we applied the semantic similarity and particularity measures on all the groups of homolog genes from the the HomoloGene database. This approach aims to identify systematically homologues expected to be similar and having also particular functions.

In all these cases, we used the GOSemSim R package to compute Lin's similarity and to provide IC tables used in the computation of the IC-based particularity [50]. We used a personal implementation of Wang's similarity and the corresponding SV used in SV-based particularity computation.

### Case 1: *Saccharomyces cerevisiae* tryptophan degradation

We first tested our approach on the example chosen by Wang [22]: *Saccharomyces Cerevisiae* tryptophan degradation [51]. We computed the semantic similarity according to Wang's method (Table S1) using the most recent version of annotation data available (August 2013 versions of GOA and GO).

Wang's conclusions remained true: we can still distinguish the three groups of genes involved in the three main steps of tryptophan degradation. Similarity values for the group [ARO8, ARO9] involved in the first step were 0.92. Similar results were observed for the group [ARO10, PDC6, PDC5, PDC1] involved in the second step and for the group [SFA1, ADH5, ADH4, ADH3, ADH2, ADH1] involved in the last step. The similarities measured between genes of 2 different groups (“inter-group measures”) were greater than in Wang's original study but remained lower than the intra-group comparison measures. We found the same three groups as Wang. These groups are biologically relevant because they are involved in the three steps of *Saccharomyces cerevisiae* tryptophan degradation pathway. To obtain these groups, Wang used a threshold of 0.770 in 2007. We used a threshold of 0.745.

We completed the previous results with the measures of semantic particularity, using Wang's Semantic Value as informativeness (Table S2). The highest particularity values were between genes from different groups which is consistent with the analysis of the semantic similarity values.

**Figure 2:**
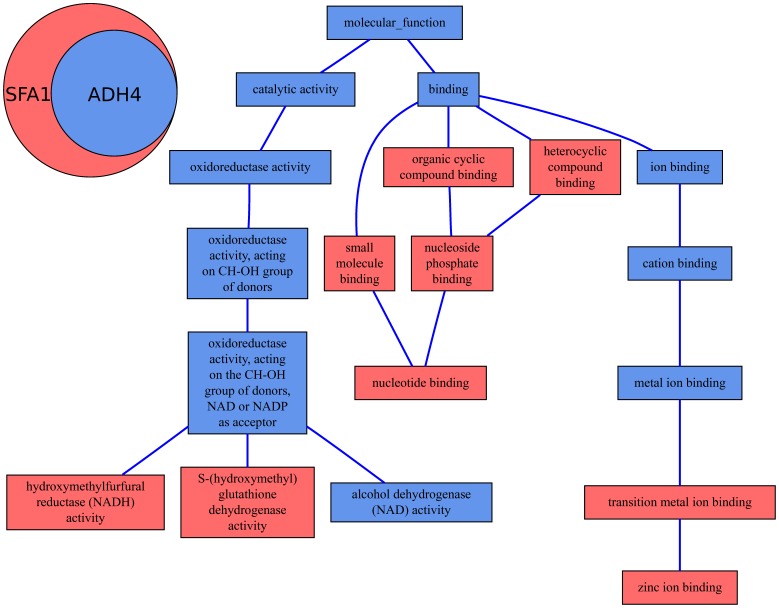
Representation of ADH4 and SFA1 *Saccharomyces cerevisiae* annotations. The particularity of 0.388 for SFA1 compared to ADH4 is explained notably by the term “nucleotide binding”, to which the closest ancestor with ADH4 annotations is at a distance of three edges. The other red terms are also responsible for this particularity.

Our approach also identified a characteristic of the compared genes that the similarity ignored. Indeed, some of the genes belonging to the same group have also some particular functions (i.e. high similarity and relatively high particularity). For example, all the genes of the third group are similar. However, Table S2 shows that all the genes of this group have a high particularity value compared to ADH4. Notably, the similarity between SFA1 and ADH4 was 0.745 and SFA1 particularity was 0.388 whereas most of the other intra-group particularity values in this group were zero or close to zero. Figure 2 presents the distribution of GO annotations between genes ADH4 and SFA1. It shows that the observed particularity value is mostly related to SFA1-specific nucleotide binding function. So, two genes can be similar while at least one of them has some particular functions.

The similarity values show that Wang results are still valid. We also identified a benefit of using a particularity measure in addition to a similarity measure for identifying particular functions between similar genes.

### Case 2: *Homo sapiens* aquaporin-mediated transport

In the previous case, we found an example of a relatively high particularity value between similar genes. In this second case, we aim to study a larger dataset in order to determine the frequency and the importance of this situation. We used a dataset composed by 51 well-annotated human genes involved in the aquaporin-mediated transport pathway for *Homo sapiens*. We used the list of all involved genes provided by the Reactome database [52]. In continuity with the first case, we computed the Wang similarity and S-Value-based particularities for each pair of genes of this list. As the Human annotation database is one of the most comprehensive, we also duplicating the study using Lin's measure as an IC-based similarity, and IC as a value of GO term informativeness for our specificity. All the results are available in File S1. Tables 1, 2 and 3 present the average, standard deviation, minimum and maximum values of particularity measured in this study for each branch of GO. We classified these statistics in 20 similarity categories containing all the comparison results ranging from sim = 0.5 to sim = 0.999 with steps of sim = 0.025.

**Table 1:** Particularity value statistics in 20 similarity values ranges from case 2 - BP measures.

BP	S-value-based particularity				IC-based particularity			
Similarity	Average	Std dev.	Min	Max	Average	Std dev.	Min	Max
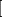 0.5–0.524 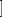	0.401	0.2	0.013	0.844	0.562	0.223	0	0.904
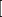 0.525–0.549 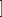	0.386	0.174	0	0.794	0.532	0.284	0	0.89
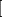 0.55–0.574 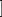	0.347	0.199	0	0.707	0.497	0.244	0	0.886
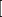 0.575–0.599 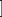	0.352	0.198	0	0.798	0.502	0.241	0	0.895
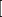 0.6–0.624 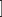	0.315	0.203	0	0.671	0.495	0.208	0	0.794
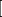 0.625–0.649 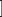	0.292	0.145	0	0.629	0.437	0.25	0	0.882
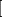 0.65–0.674 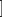	0.299	0.162	0	0.615	0.439	0.258	0	0.876
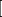 0.675–0.699 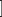	0.229	0.15	0	0.529	0.451	0.216	0.039	0.839
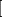 0.7–0.724 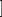	0.228	0.166	0	0.631	0.403	0.239	0	0.859
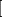 0.725–0.749 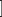	0.22	0.145	0	0.501	0.35	0.233	0	0.727
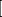 0.75–0.774 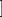	0.202	0.108	0	0.482	0.403	0.207	0	0.775
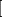 0.775–0.799 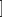	0.178	0.118	0	0.563	0.319	0.222	0	0.671
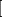 0.8–0.824 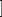	0.177	0.106	0	0.418	0.31	0.209	0.043	0.646
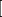 0.825–0.849 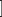	0.125	0.071	0	0.327	0.258	0.184	0	0.589
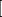 0.85–0.874 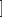	0.105	0.131	0	0.418	0.201	0.136	0	0.625
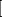 0.875–0.899 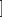	0.061	0.066	0	0.248	0.179	0.123	0	0.651
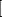 0.9–0.924 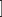	0.039	0.061	0	0.211	0.207	0.156	0	0.614
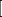 0.925–0.949 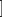	0.041	0.067	0	0.248	0.193	0.181	0	0.572
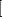 0.95–0.974 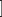	0.032	0.041	0	0.111	0.099	0.076	0	0.196
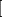 0.975–0.999 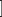	0.005	0.006	0	0.015	0.077	0.152	0	0.519

This table gives the average, standard deviation, minimum and maximum particularity value for the BP comparisons of the case 2. The 20 categories contain all the results that range from a similarity of 0.5 to 0.999 with steps of 0.025.

**Table 2:** Particularity value statistics in 20 similarity values ranges from case 2 - MF measures.

MF	S-value-based particularity				IC-based particularity			
Similarity	Average	Std dev.	Min	Max	Average	Std dev.	Min	Max
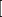 0.5–0.524 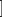	0.341	0.26	0	0.798	0.494	0.162	0.296	0.701
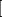 0.525–0.549 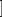	0.35	0.219	0	0.818	0.429	0.212	0	0.703
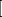 0.55–0.574 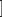	0.364	0.32	0	0.731	0.422	0.265	0	0.849
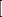 0.575–0.599 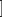	0.382	0.265	0	0.694	0.378	0.148	0.125	0.591
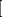 0.6–0.624 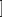	0.242	0.079	0.132	0.47	0.397	0.205	0	0.81
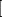 0.625–0.649 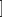	0.207	0.113	0	0.531	0.302	0.145	0.158	0.475
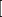 0.65–0.674 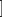	0.281	0.106	0.117	0.482	0.609	0.137	0.13	0.806
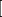 0.675–0.699 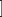	0.223	0.181	0	0.562	0.453	0.249	0	0.763
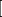 0.7–0.724 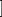	0.26	0.267	0	0.564	0.389	0.248	0	0.806
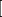 0.725–0.749 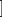	0.179	0.176	0	0.482	0.419	0.211	0	0.763
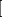 0.75–0.774 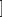	0.171	0.177	0	0.371	0.315	0.216	0	0.643
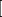 0.775–0.799 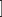	0.125	0.167	0	0.482	0.33	0.241	0	0.777
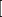 0.8–0.824 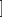	0.063	0.056	0	0.137	0.239	0.218	0	0.574
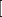 0.825–0.849 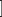	0.119	0.13	0	0.415	0.316	0.222	0	0.574
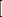 0.85–0.874 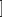	0.041	0.036	0	0.116	0.266	0.175	0	0.531
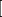 0.875–0.899 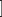	0.045	0.05	0	0.126	0.179	0.093	0.086	0.272
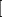 0.9–0.924 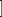	0.024	0.025	0	0.055	0.163	0.153	0	0.388
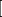 0.925–0.949 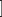	0.02	0.026	0	0.086	0.09	0.107	0	0.272
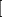 0.95–0.974 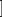	0.005	0.007	0	0.023	-	-	-	-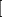 0.975–0.999 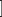	-	-	-	-	-	-	-	-

This table gives the average, standard deviation, minimum and maximum particularity value for the MF comparisons of the case 2. The 20 categories contain all the results that range from a similarity of 0.5 to 0.999 with steps of 0.025. “-” value denotes an empty category.

**Table 3:** Particularity value statistics in 20 similarity values ranges from case 2 - CC measures.

CC	S-value-based particularity				IC-based particularity			
Similarity	Average	Std dev.	Min	Max	Average	Std dev.	Min	Max
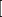 0.5–0.524 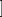	0.353	0.233	0	0.846	0.621	0.244	0	0.911
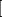 0.525–0.549 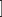	0.36	0.214	0	0.819	0.707	0.15	0.185	0.977
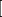 0.55–0.574 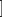	0.33	0.187	0	0.799	0.64	0.202	0	0.897
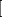 0.575–0.599 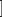	0.341	0.185	0	0.752	0.613	0.194	0	0.896
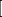 0.6–0.624 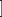	0.317	0.183	0	0.754	0.621	0.165	0	0.888
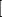 0.625–0.649 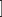	0.268	0.18	0	0.706	0.592	0.207	0	0.852
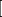 0.65–0.674 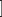	0.28	0.177	0	0.656	0.553	0.227	0	0.888
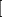 0.675–0.699 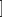	0.24	0.177	0	0.583	0.495	0.241	0	0.845
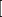 0.7–0.724 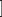	0.13	0.159	0	0.543	0.466	0.24	0	0.825
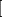 0.725–0.749 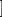	0.196	0.151	0	0.579	0.428	0.268	0	0.82
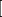 0.75–0.774 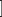	0.134	0.122	0	0.484	0.383	0.246	0	0.819
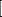 0.775–0.799 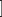	0.15	0.127	0	0.489	0.391	0.267	0	0.768
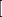 0.8–0.824 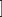	0.144	0.093	0	0.269	0.19	0.187	0	0.625
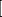 0.825–0.849 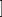	0.133	0.123	0	0.421	0.352	0.231	0	0.73
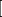 0.85–0.874 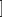	0.146	0.152	0	0.373	0.255	0.216	0	0.624
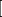 0.875–0.899 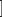	0.051	0.051	0	0.11	0.145	0.152	0	0.381
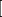 0.9–0.924 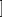	0.067	0.085	0	0.269	0.095	0.095	0	0.189
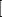 0.925–0.949 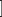	-	-	-	-	-	-	-	-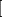 0.95–0.974 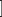	-	-	-	-	0.131	0.131	0	0.262
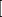 0.975–0.999 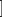	0.012	0.012	0	0.024	0.049	0.049	0	0.098

This table gives the average, standard deviation, minimum and maximum particularity value for the CC comparisons of the case 2. The 20 categories contain all the results that range from a similarity of 0.5 to 0.999 with steps of 0.025. “-” value denotes an empty category.

The relatively high particularity between similar genes that we observed in case 1 is confirmed in this case 2. In each 20 categories in the human aquaporin-mediated transport pathway, some of the genes have an important particularity compared to the others. Again, these genes cannot be identified using only a similarity measure.

**Figure 3:**
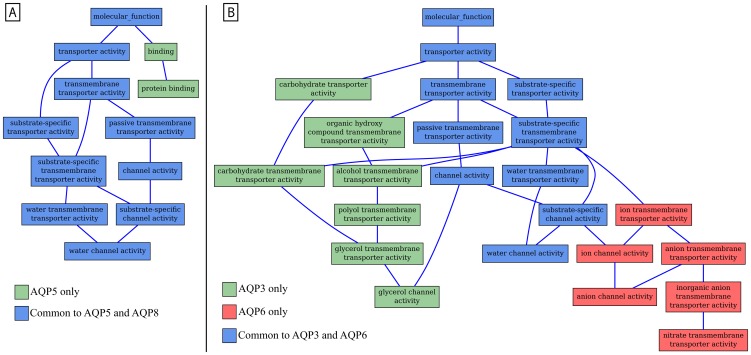
MF annotations of two couples of human aquaporins. Part A: AQP8 and AQP5 share most of their annotations. Part B: AQP6 and AQP3 share numerous molecular functions, but each gene also have particular functions.

Figure 3 illustrates this case giving the MF annotation graph of two couples of genes: AQP8 and AQP5 in part A and AQP6 and AQP3 in part B. The corresponding similarity and particularity values are presented in Table 4. The two couples have close similarity values regardless the method used but they show a very different particularity profile, with much higher particularities between AQP6 and AQP3 than between AQP8 and AQP5. The two distinct informativeness measures used to compute the particularity led to the same conclusion. The same phenomenon can be observed in the 20 categories of similar genes.

**Table 4:** Similarity and particularity values of two couples of genes from case 2.

SV-based		AQP6	AQP3	IC-based		AQP6	AQP3
2*Sim	AQP6	1	0.696	2*Sim	AQP6	1	0.81
	AQP3		1		AQP3		1
2*Par	AQP6	0	0.247	2*Par	AQP6	0	0.531
	AQP3	0.415	0		AQP3	0.388	0

SV-based		AQP8	AQP5	IC-based		AQP8	AQP5
2*Sim	AQP8	1	0.704	2*Sim	AQP8	1	0.8
	AQP5		1		AQP5		1
2*Par	AQP8	0	0	2*Par	AQP8	0	0
	AQP5	0.19	0		AQP5	0.13	0

The similarity between AQP6 and AQP3 is very close to the similarity between AQP8 and AQP5 regardless the method used (SV or IC-based). However, the particularity profile obtained for each couple is very different. Again, the SV-based and IC-based methods led to the same conclusion.

These results confirm that among similar genes, some also have some particular functions, and show that this situation can be observed throughout the full range of similarity values. Therefore, the situation described in the first use case was not an isolated case.

### Case 3: Homologs comparison

The previous cases focused on the similarity and particularity of different genes in a same pathway. In this third case, we compared homolog genes across different species. IC-based methods cannot be used in this cross-species context. To investigate the frequency of similar homolog genes and the frequency of homolog genes having particular functions, we computed Wang's semantic similarity and SV-based particularities for each group of the HomoloGene database. The August 2013 version of this database contained 43,074 groups of homolog genes. Each group contained from 2 to 839 genes (average: 6.02, standard deviation: 7.46). We computed all the 5,531,994 intra-group similarity and particularity measures. Table 5 categorizes the comparisons according to the number of annotated genes.

**Table 5:** Similarity and particularity pattern in pairwise comparisons on homolog genes in the HomoloGene database.

Branch of GO	BP	MF	CC	All
Number of comparisons	1,843,998	1,843,998	1,843,998	5,531,994
Only one gene is annotated	511,899	574,815	581,819	1,668,533
No annotated gene	939,010	823,444	887,419	2,649,873
Two genes annotated	393,089	445,739	374,760	1,213,588
Sim 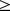 0.5; All Par<0.5	287,288	396,412	314,572	998,272
Sim 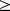 0.5; One Par 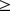 0.5	39,312	20,754	32,531	92,597
Sim 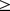 0.5; Two Spe 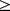 0.5	410	91	54	555
Sim<0.5	66,079	28,482	27,603	122,164

The five last lines refer to valid comparisons where the two genes were annotated.

To be valid, a comparison has to involve two annotated genes. Overall, 21.94% of the comparisons were valid. For BP, CC and MF, we used the number of valid comparisons as the baseline to analyze the different configurations of similarity and particularity. We focused on these valid comparisons and found that 89.93% of them had a similarity greater than or equal to 0.5. In 82.26%, the genes were similar and had particularities lower than 0.5. Although there were differences between BP, MF and CC, on the whole HomoloGene database, the particularity values allowed us to identify 7.63% of the valid comparisons that denote similar genes, one of these genes having a particularity greater than 0.5.

As an example illustrating the results, we analyzed the comparisons of the GO molecular functions associated to Exportin-5 orthologs for 9 species (Table S3). 27 of the 36 comparisons (75%) involved pairs of genes with a similarity greater than 0.5. 12 of these 27 comparisons involved similar pairs of genes, one of them having a particularity greater than 0.3 (mostly for *Canis canis* and *Drosophila melanogaster*). Among these, five comparisons involving *Canis canis* resulted in a similarity value over 0.5 and one particularity value over 0.5. The remaining 9 of the 36 comparisons involved genes with a similarity lower than 0.5 and particularities greater than 0.5 (mostly for *Arabidopsis thaliana* and one for *Canis canis*).

Altogether, the case 3 results showed that ortholog genes were, as expected, mostly similar. We have also demonstrated that some of them may have high particularity values that denote particular functions. Last, some orthologs may have diverged to present a low similarity and high particularities.

## Discussion

### Semantic particularity

Semantic similarity measures have been extensively used for comparing genes and gene sets [19] but they only tell a part of the story. Similarity is symmetric. It decreases slowly as the number of gene-particular annotations increases. However, similarity alone does not indicate which gene has some particular functions and does not even reveal these particular functions. There is a need for a measure to qualify this particularity (does gene1 have some particular functions compared to gene2, even if gene1 and gene2 are similar?) and to quantify these respective differences (what is the importance of gene1's particular functions compared to gene2?). Simple comparisons of the sets of terms annotating two genes, such as Venn diagram representations, give an initial picture of each gene's particularity. However, this approach is biased due to the relations between the terms of an ontology. Like for similarity, measuring particularity has to take semantics into account. Diaz-Diaz et al. proposed a semantic approach to compute a dissimilarity measure in order to evaluate the functional coherence of entire gene sets [46]. The dissimilarity of two terms is obtained by measuring a distance in edges in the GO graph and weighting the result with the depth of the considered terms, as in Wu and Palmer's similarity measure [43]. This notion of dissimilarity is therefore strongly related to similarity and does not provide a way to compute the particularity as we defined earlier (high dissimilarity indicates low similarity, and vice versa). However, the two categories of similarity measures, i.e. “edge-based” and “node-based”, can be used for this purpose. Each approach has its drawbacks [33]. Edge-based methods are biased because the GO terms are not homogeneously distributed across the tree, while node-based methods that use an IC value are dependent on a specific annotation corpus, which puts a limit on their use for cross-species comparisons. In cross-species studies, it is impossible to compare IC values relying on term frequencies obtained from different corpora. Using a global corpus instead, such as the UniprotKB GOA table is biased in favor of the most studied functions in the most studied species. Therefore, graph-based approaches relying on the distance to the root are more appropriate in such situation.

We based our semantic particularity measure on the concept of informativeness of GO terms. This informativeness can either be an Information Content (IC) [29]–[31], [33], [34] value or a Semantic Value (SV) [22]. The choice between these two alternatives depends on the data to compare. IC is preferred to compare genes from a same species when an important annotation corpus is available for this species. SV is preferred to compare genes from different species or genes from a same species without an important annotation corpus. Therefore, we advise to use a combination of either IC-based or of SV-based similarity and particularity measures when computing profiles based on similarity and particularity values.

The interpretation of the similarity and particularity values depends on the number and quality of the annotations. If at least one of two genes has few annotations, the similarity and particularity values will suffer from a lack of precision (the values are sensitive to the addition of new annotations) regardless of their accuracy.

Furthermore, annotations are associated with different Evidence Codes (EC), ranging from automatic inference to experimental validation. The biological interpretation of similarity and particularity values is more convincing when their computation refers to experimentally-confirmed annotations. However, electronically-inferred annotations may still yield valid similarity and particularity values. As the GO consortium recommends against using EC as a measure of quality of the annotation [53], we did not use them to weight the similarity and particularity values. However, we paid attention to this aspect when interpreting the results of our case studies. Our approach consisted in comparing two genes using a tuple of one symmetric similarity value and the two particularity values. Having high similarity and low particularities for two genes indicates that these genes globally have the same characteristics in the compared domain (BP, MF or CC) and none of them has any major additional particularity. Conversely, a low similarity and high particularities between two genes indicates that these genes are different in the compared domain. Furthermore, among highly similar genes, finding that one gene has also a high particularity value allows to identify additional features for this gene not present in the other one despite their high similarity. This contributed to a more accurate analysis than using similarity alone by distinguishing interesting sub-groups of features with close similarity values.

### Case studies: benefits of the semantic particularity

Particularity refined the similarity-based analysis by identifying some couples of similar genes with important particularities. All three use cases illustrated this point in intra-species or in cross-species.

In the first case study on the *Saccharomyces Cerevisiae* tryptophan degradation pathway, SFA1 and ADH4 had similarity values close to those of the other genes of the same sub-group. However, SFA1 and to a lesser extent all the other genes that catalyze the same reaction had some particular functions compared to ADH4. Consequently, it is possible that two similar genes also have some particular functions (i.e. high similarity and relatively high particularity). The particularity is not systematically inversely proportional to the similarity. Moreover, some of these these atypical cases may be of biological interest.

We have gone further in the case 2, comparing 51 genes that belong to a same human pathway. With this case, we wanted to see three things. First, we wanted to know whether the observations made in the first case remained true on a bigger example. They did. Then, we wanted to assess the effect of the kind of informativeness used. Semantic value and information content gave different semantic similarity and particularity values, but they leaded to the same conclusions. Consequently, the choice of this method only depends on the data we want to compare. IC can be used as an informativeness measure if the data are relative to one single species and if this species is sufficiently annotated to offer a meaningful corpus. Otherwise, the best informativeness measure may be the semantic value. Last, we wanted to assess our conclusions on the three branches of Gene Ontology. Concerning this point, we obtained high particularity values between similar genes regarding any branch of GO.

The third case showed comparisons of ortholog genes that also resulted in interesting sub-cases with high-similarity profiles. As suspected, the results confirmed that ortholog genes are mostly similar. Moreover, particularity measures made it possible to observe that among the pairs of similar genes, some are composed of at least one gene having also an important particularity. Indeed, among the 1,213,588 valid comparisons across the whole HomoloGene database, we identified 93,152 (7.68%) comparisons for which the genes were similar, but at least one of them had an important particularity, denoting some particular function(s). This confirm the observations made in the cases 1 and 2. These 7.68% of valid comparisons resulting in the identification of genes having some particular features, which however have enough common GO annotation to remain similar are biologically very interesting. This demonstrates the benefit s of using the semantic particularity measure in addition to semantic similarity.

In the third case, we developed the Exportin-5 example to illustrate the limitations of the semantic similarity measures. The results of a similarity measure did not reflect that the amount of particular functions while comparing the human gene to the drosophila ortholog (“tRNA binding” and four of its ancestors are human-specific) is greater than while comparing it to the rat ortholog (only “protein binding” is human-specific). The particularity measure showed that the human and drosophila Exportin-5 orthologs are not only similar, but that some quantifiable features are in reality very specific to the human gene. Furthermore, the high particularity of these orthologs is consistent with the results of Shibata et al., who demonstrated that Exportin-5 orthologs are functionally divergent among species [49].

### Interpretation of similarity and particularity values

The case studies showed that combining similarity and particularity makes it possible to identify some genes' particular functions that cannot be distinguished using similarity only. These particular functions may be the result of a real biological difference, a default of annotation, or a combination of both. If we suspect a default of annotation, the results should be interpreted carefully until the annotations are improved.

In the case 3, the number of annotations vary between the compared orthologs. On the one hand, the results can reflect a real particularity of function for some genes. On the other hand, the high particularity of a gene can be the result of a lack of annotations of the other gene. For example, when comparing MF annotations for hsa and ath orthologs of Exportin-5, we observed very high particularities for both species (respectively 0.641 and 0.871). We consider these results to be relevant, as the genes of both species are well annotated (11 annotations in the expanded set of hsa, 18 annotations in the expanded set of ath). Conversely, care is warranted when interpreting the particularity of hsa over Canis canis (cca). For these species, sim(hsa, cca) = 0.428, spe(hsa, cca) = 0.611 and spe(cca, hsa) = 0. However, the expanded set of annotations for the cca ortholog had only 4 terms compared to 11 for hsa. In this case, the high particularity of hsa could be attributed to the lack of cca annotations.

### Synthesis

We showed that gene set analysis can be improved by considering gene-set particularities in addition to their similarity. We proposed a set of formal properties and a new GO semantic measure to compute gene-set particularity. We first showed that particularity is a useful complement to similarity for comparing gene sets, making it possible to detect similar gene sets for which one of the sets also had some particular functions, and to identify these functions. We also showed that using particularity also improves gene clustering. Our particularity measure relies on the informativeness of GO terms. This informativeness of a term can be its Information Content or its Semantic Value. In this paper, we combined our particularity measure with a similarity measure to compare genes annotated GO terms, but this same principle can be generalized to other ontologies.

## Supporting Information

File S1 **Complete results for the case 2 about** ***Homo sapiens*** **aquaporin-mediated transport.**
(ZIP)

Table S1 **Semantic similarity values between genes involved in the** ***Saccharomyces cerevisiae*** **tryptophan degradation pathway.** Color gradient according to similarity value (0 = white, 1 = blue). The given numbers of annotations (“Annots”) consider the GO terms that annotate directly the genes and their ancestors.
(TIF)

Table S2 **Semantic particularity values between genes involved in the** ***Saccharomyces cerevisiae*** **tryptophan degradation pathway.** Color gradient according to particularity value (0 = white, 1 = red or green). If Par(gene1, gene2) is displayed in green, Par(gene2, gene1) is displayed in red. The value contained in a cell is the particularity of the gene displayed at its row header compared to the gene displayed at its column header. For example, Par(ARO10, ARO8) = 0.62 and Par(ARO8, ARO10) = 0.506. The given numbers of annotations (“Annots”) consider the GO terms that annotate directly the genes and their ancestors.
(TIF)

Table S3 **Semantic similarity and particularity values between Exportin-5 orthologs in 9 species.** Color gradient according to similarity value (0 = white, 1 = blue) and particularity values (0 = white, 1 = red or green). If Par(gene1, gene2) is displayed in green, Par(gene2, gene1) is displayed in red. The value contained in a cell is the particularity of the gene displayed at its row header compared to the gene displayed at its column header. The given numbers of annotations (#Annot) consider the total number of GO terms that annotate the genes either directly or indirectly).
(TIF)
