# Supplementary figures and images for: Semantic Particularity Measure for Functional Characterization of Gene Sets Using Gene Ontology
Source: PLoS One. 2014 Jan 28;9(1):e86525. doi: 10.1371/journal.pone.0086525 (PMC3904913; doi:10.1371/journal.pone.0086525)

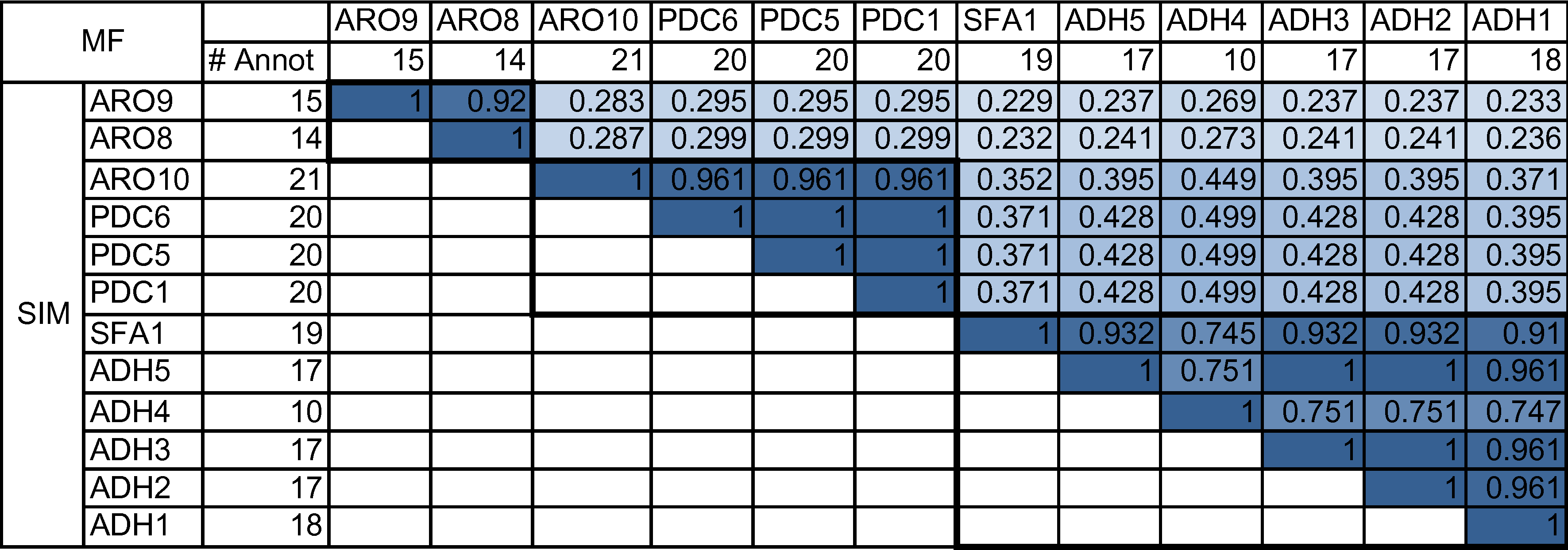

Supplement: Table S1 — Semantic similarity values between genes involved in the Saccharomyces cerevisiae tryptophan degradation pathway. Color gradient according to similarity value (0 = white, 1 = blue). The given numbers of annotations (“Annots”) consider the GO terms that annotate directly the genes and their ancestors. (TIF) [file pone.0086525.s002.tif]

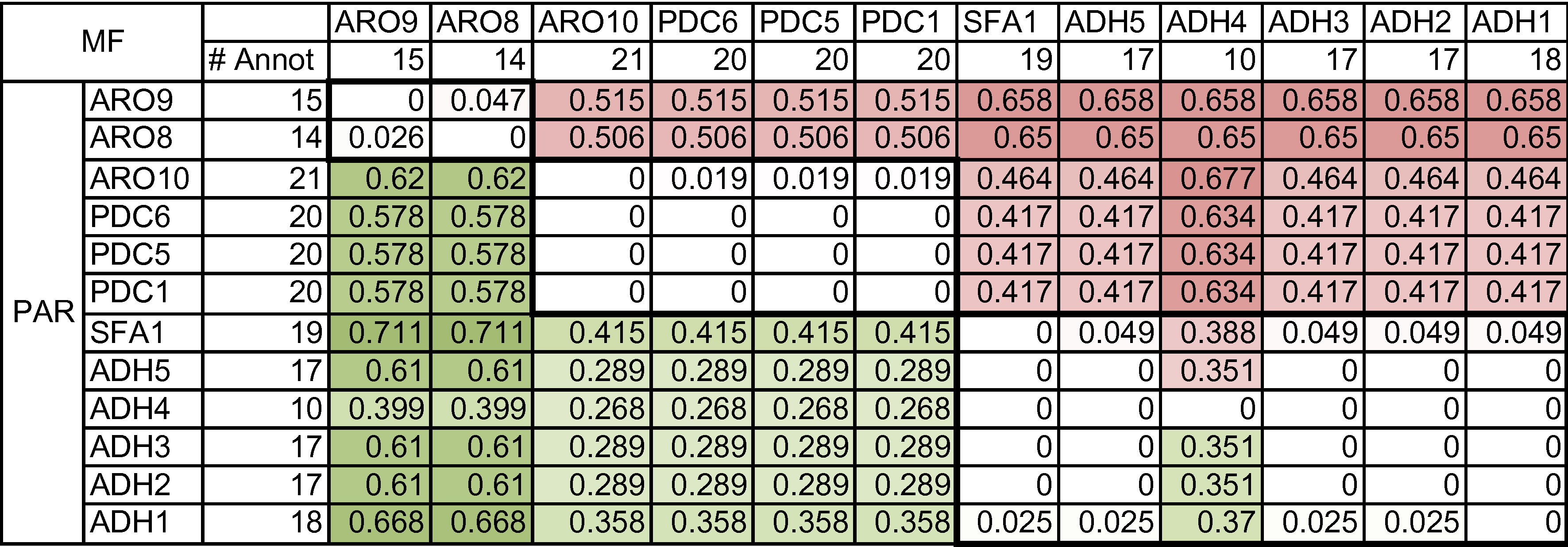

Supplement: Table S2 — Semantic particularity values between genes involved in the Saccharomyces cerevisiae tryptophan degradation pathway. Color gradient according to particularity value (0 = white, 1 = red or green). If Par(gene1, gene2) is displayed in green, Par(gene2, gene1) is displayed in red. The value contained in a cell is the particularity of the gene displayed at its row header compared to the gene displayed at its column header. For example, Par(ARO10, ARO8) = 0.62 and Par(ARO8, ARO10) = 0.506. The given numbers of annotations (“Annots”) consider the GO terms that annotate directly the genes and their ancestors. (TIF) [file pone.0086525.s003.tif]

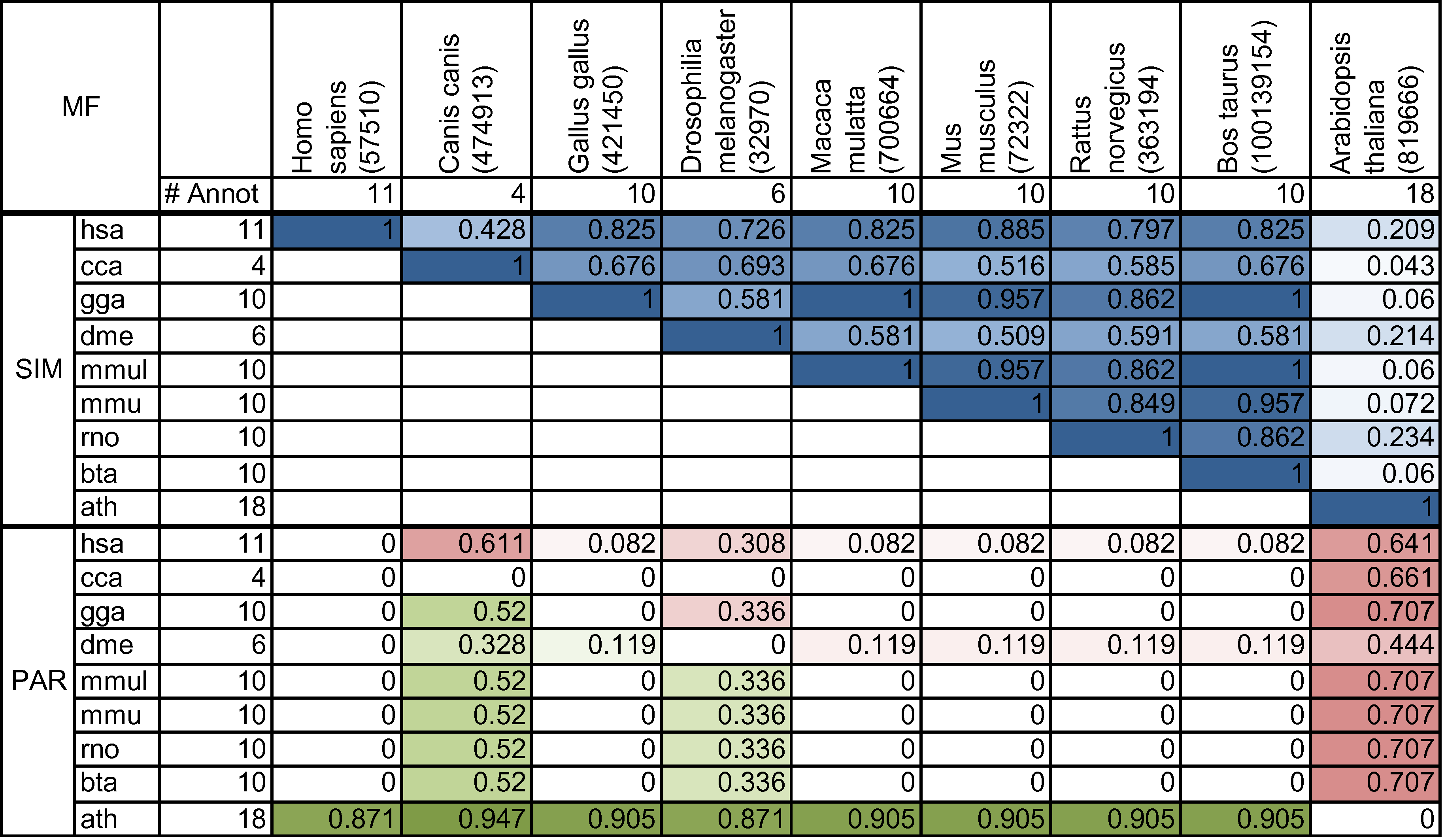

Supplement: Table S3 — Semantic similarity and particularity values between Exportin-5 orthologs in 9 species. Color gradient according to similarity value (0 = white, 1 = blue) and particularity values (0 = white, 1 = red or green). If Par(gene1, gene2) is displayed in green, Par(gene2, gene1) is displayed in red. The value contained in a cell is the particularity of the gene displayed at its row header compared to the gene displayed at its column header. The given numbers of annotations (#Annot) consider the total number of GO terms that annotate the genes either directly or indirectly). (TIF) [file pone.0086525.s004.tif]
